# What Drives Seasonal Mat Dynamics in Eutrophic Lake: Allelopathy or Environmental Gradients?

**DOI:** 10.1002/ece3.74213

**Published:** 2026-08-26

**Authors:** Gostyńska Julia, Gąbka Maciej, Pankiewicz Radosław, Messyasz Beata

**Affiliations:** ^1^ Department of Hydrobiology, Faculty of Biology Adam Mickiewicz University Poznań Poland; ^2^ Department of Environmental Physicochemistry, Faculty of Chemistry Adam Mickiewicz University Poznań Poland

**Keywords:** adaptation strategy, *Cladophora glomerata*, *Lemna minor*, phenolic compounds, temporal niche partitioning

## Abstract

Eutrophication of aquatic ecosystems promotes the occurrence of filamentous macroalgae and free‐floating macrophytes that form dense surface mat in the littoral zone of shallow water bodies. The structure of these communities is shaped by environmental gradients, species features, and tolerance to competition‐related stressors. While laboratory studies show allelopathic potential of these primary producers, field‐based evidence integrating chemical interactions with environmental gradients is still poorly understood. We conducted a two‐season field study in eutrophic Lake Oporzyńskie to assess the relative importance of allelopathy and environmental gradients in regulating community dynamics of *C. glomerata* and *L. minor* . We measured the phenolic content in water and species extracts, physicochemical water parameters and mat size, analyzing the associations using RDA, variance partitioning, and GAM. Variance partitioning showed that phenolics and physicochemical parameters jointly explained more variation in mat size (adjusted *R* ^2^ = 0.446) than either fraction alone, with phenolics contributing a markedly larger unique fraction (*R* ^2^ = 0.337) than physicochemical parameters (*R* ^2^ = 0.060). RDA identified phenolic content in water as the strongest predictor of mat size, with an asymmetric relationship, positive for *C. glomerata* and negative for *L. minor* , consistent with an allelopathic advantage for the macroalga, which also had higher phenolic content in its own extract. GAM analyses confirmed this pattern and revealed contrasting, taxon‐specific response curves to phenolic content, pH, dissolved oxygen, and TDS. This was associated with temporal niche partitioning, with *C. glomerata* dominating mid‐summer and *L. minor* expanding in late summer and autumn. Our findings suggest that coexistence of these species reflects the combined action of allelopathic interactions and environmental gradients generating species‐specific temporal windows of dominance. This integrated approach highlights the need to jointly consider chemical and environmental drivers when studying plant community dynamics in aquatic ecosystems.

## Graphical abstract

A two‐season field study of eutrophic Lake Oporzyńskie showed that phenolic compounds and water physicochemistry jointly, but unevenly, drive the mat dynamics of *Cladophora glomerata* and *Lemna minor* , with phenolics explaining a much larger part of the variability in mat size than physicochemical parameters. The content of phenolic compounds in water proved to be the strongest predictor, showing an asymmetric effect—positive for *C. glomerata* and negative for *L. minor* —which is consistent with the allelopathic predominance of the macroalga. These chemical and environmental drivers together generated temporal niche partitioning, with a dominant mat of *C. glomerata* in mid‐summer and *L. minor* expanding later in the season, suggesting that coexistence results from the combined action of allelopathy and environmental gradients rather than the action of either alone.

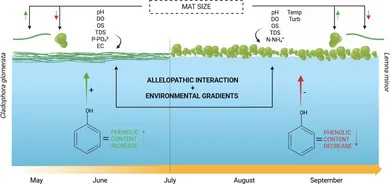

## 1 Introduction

Eutrophication of aquatic ecosystems, characterized by excessive enrichment of nutrients, primarily nitrogen and phosphorus, is one of the most serious global environmental challenges (Akinnawo 2023; Smith and Schindler 2009). One of the visible symptoms of this process in the littoral zone of shallow aquatic ecosystems is the occurrence of macroalgae (Vadeboncoeur et al. 2021; Valiela et al. 1997). *Cladophora glomerata* (L.) Kütz, a common filamentous macroalga worldwide, dominates eutrophic freshwater ecosystems, where its biomass increases with rising levels of nitrate and orthophosphate availability (Dodds 1991; Higgins et al. 2008) and under alkaline conditions (Pikosz and Messyasz 2016).

The occurrence and abundance of *C. glomerata* in bodies of water are also regulated by the following environmental gradients: depth, temperature, light, dissolved oxygen concentration, nutrient, hydrological characteristics of the reservoir, substrate properties to which it is attached, and interactions with other organisms (Dodds 1991; Michalak and Messyasz 2021; Parker and Maberly 2000; Vadeboncoeur et al. 2021). This macroalga acts as an ecological engineer, modifying the functioning of aquatic ecosystems by strongly absorbing nutrients during growth, reducing water transparency, changing dissolved oxygen dynamics by forming a dense mat on the water surface, increasing water pH as a result of photosynthetic carbon uptake, and providing substrate for epiphytic colonization (Higgins et al. 2008; Wu et al. 2024; Zulkifly et al. 2013). Macroalgae exhibit a variety of allelopathic and non‐allelopathic interactions with phytoplankton, other macroalgae and macrophytes in aquatic ecosystems, which may lead to a decline of these communities (Gross 2003; Irfanullah and Moss 2005; Pikosz et al. 2019). *C. glomerata* synthesizes secondary metabolites, including polyphenols, flavonoids, and phenolic acids (Korzeniowska et al. 2020; Nutautaitė et al. 2022). The production of these compounds increases under competitive pressure from other primary producers, potentially enhancing the ecological dominance and allelopathic effects of *Cladophora*, thereby facilitating extensive mat formation (Fabrowska et al. 2018).

In addition to macroalgae in the littoral zone of shallow eutrophic lakes, macrophytes are a fundamental structural component, playing a key role in primary productivity, nutrient cycling, sediment stabilization, and providing habitat and food resources for diverse communities of aquatic organisms (Carpenter and Lodge 1986; Thomaz and Cunha 2010). Among the various functional forms of macrophytes, free‐floating species can occupy a similar ecological niche to filamentous macroalgae in aquatic ecosystems. The free‐floating macrophytes can also modify ecosystem functioning by effectively removing phosphates and nitrogen (Hendriks et al. 2023) and altering dissolved oxygen dynamics by producing oxygen through photosynthesis (Sender and Różańska‐Boczula 2024), but can also create hypoxic conditions under a dense mat by limiting gas exchange and photosynthesis in deeper water layers (Caraco and Cole 2002; Ceschin et al. 2020). This mat also reduces light penetration, which inhibits the growth of submerged vegetation, promoting a persistent vegetated state in shallow, nutrient‐rich bodies of water (Scheffer et al. 2003; Szabó et al. 2005).

According to our field observations in shallow eutrophic Lake Oporzyńskie in the western region of Poland, the filamentous macroalga *C. glomerata* co‐occurred with the free‐floating macrophyte *L. minor* in the growing season, both forming dense mats on the water surface in the littoral zone. *Lemna minor* L. is one example of free‐floating macrophytes widely distributed in freshwater ecosystems worldwide (Landolt 1986), which can also release secondary metabolites with allelopathic effects, influencing biological processes in other organisms (Bich and Kato‐Noguchi 2012; Gostyńska et al. 2022). Our previous review study demonstrated that both *C. glomerata* and *L. minor* exhibit allelopathic potential through the production of phenolic compounds with higher content in *C. glomerata* extract, highlighting the important role of allelopathy in interspecies interactions (Gostyńska et al. 2022). However, the study left unanswered whether allelopathic compounds (phenolic compounds) are the only factor regulating macroalga and macrophyte community dynamics in the aquatic ecosystem. It is common knowledge that the structure and size of aquatic plant communities are also modulated by environmental features, including temperature, pH, nutrients, and trophic status, as well as species‐specific traits and tolerance to environmental stressors related to competition (Bornette and Puijalon 2011; Lacoul and Freedman 2006).

Most existing studies on allelopathic interactions between aquatic plants have been conducted under controlled laboratory conditions, where environmental variables can be isolated (Gross et al. 2007; Hilt et al. 2006). While such studies provide significant mechanistic insights, they may not reflect the complexity of natural systems where multiple environmental gradients fluctuate. Comprehensive field‐based studies that integrate measurements of allelopathic compounds with environmental variables across seasonal cycles are needed to distinguish the relative contributions of chemical compounds versus physicochemical conditions in shaping species interactions and community structure. Understanding how competing species coexist in shared habitats under multiple interacting factors remains a fundamental question in aquatic ecology.

To address these knowledge gaps, we conducted a comprehensive field study across two growing seasons (2022–2023) in Lake Oporzyńskie. Unlike our previous review research, this study integrated weekly measurements of phenolic content (in water and species extracts) and physicochemical water parameters to assess the relative importance of allelopathy versus environmental gradients in regulating the community dynamics of *C. glomerata* and *L. minor* . Our main aims and hypotheses are as follows:

- To assess the relationship between phenolic compounds and mat size of the macroalga and macrophyte in a freshwater ecosystem: we hypothesize that phenolic compounds are strongly correlated with mat size patterns in both studied species, with potential asymmetric effects, creating competitive advantage for *C. glomerata* . Specifically, we predict that expansion of the macroalga mat is positively associated with phenolic content in water, while macrophyte mat size is negatively associated with responses to higher environmental phenolic levels.
- To examine the influence of environmental gradients on species‐specific growth responses: we hypothesize that *C. glomerata* and *L. minor* exhibit different growth responses to environmental gradients, creating different dominance patterns for each species and potentially reducing direct competition through the secretion of phenolic compounds.

## 2 Methods

### 2.1 Study Area

The environmental work was conducted in Lake Oporzyńskie (52°55′34″ N, 17°09′34″ E), located in the western region of Poland. This shallow, eutrophic lake has a surface area of 0.15 km^2^, a mean depth of 1.2 m, and a maximum depth of 2.0 m (Pankiewicz et al. 2016; Pikosz et al. 2017). The lake is situated in a depression with steep slopes and is surrounded by agricultural fields (forests accounting for < 5%), which promotes high nutrient loading through streams (Pikosz et al. 2017).

### 2.2 Field Sampling

We conducted a weekly in situ study in Lake Oporzyńskie, starting from the first week of May until the end of September in 2022 and 2023, obtaining 21 sampling dates per year (*n* = 42 total). At each sampling date, we quantified the mat size (m^3^) of *C. glomerata* and *L. minor* on three randomly selected distinct mats in the littoral zone in an area representative of the highest mat occurrence. The mat area of each species was measured using a specially designed measuring device equipped with a 1 m wide scale, which was systematically moved across the mat surface to determine total coverage. The mat's depth was measured using a specially designed device with a 1 m long scale. The total mat size was calculated as the product of the area and depth.

The physicochemical parameters were measured using a YSI ProDSS (YSI Incorporated, USA) multiparameter probe, including pH, water temperature (°C), total dissolved solids (mg L^−1^), oxygen saturation (%), dissolved oxygen (mg L^−1^), turbidity (NTU), and electrolytic conductivity (μS cm^−1^) 10 cm below the water surface near the mat of the species studied.

The water samples for analysis of orthophosphates (mg P L^−1^), sulphates (mg L^−1^), nitrate (mg N L^−1^), and ammonium nitrogen (mg N L^−1^), were collected using a suction pump equipped with a hose (ø 10 mm) adapted to extract water into calibrated vessels from a depth of 10 cm. A 500 mL water sample was collected and stored at −10°C for further analyses. Chemical analyses were performed by means of HACH DR 2800 spectrophotometer using standard Hach methods: orthophosphate was determined using Hach Method 8048, sulphates using Hach Method 8051, nitrate using Hach Method 8171, and ammonium nitrogen using Hach Method 8038 (Hach Company 2007).

For analysis of the total phenolic content in water, 0.5 L of water was collected from a depth of 10 cm outside the macroalga and macrophyte mat into plastic containers. Water samples were ultra‐filtered to remove any microorganisms and stored at −20°C in a laboratory freezer and protected from light until analysis. Samples of *C. glomerata* and *L. minor* were randomly collected and transported at 4°C in a refrigerated container to a laboratory for analysis of the total phenolic content in extracts of these species.

### 2.3 Extraction

*C. glomerata* and *L. minor* samples were extracted using microwave‐assisted extraction (MAE). MAE process was performed using the microwave oven (Mars Xpress). In each extraction experiment 2 g of dry *C. glomerata* and *L. minor* samples were used 40°C was the extraction temperature, 2 h was the extraction time and 50 mL of ethanol was used as a solvent. The other parameters specified for MAE were power −800 W and yield—100%. The extracts obtained were collected in vials and the residual ethanol was evaporated using a vacuum evaporator (Buchi, R II) to calculate the extraction yield. Then, dried extracts were diluted with methanol to obtain the concentration of 10 mg mL^−1^. Diluted extracts were stored at −20°C and protected from light until analysis.

### 2.4 Chemicals and Reagents for Extraction

The chemicals used in this study included: ethanol (especially pure, 99.8%, POCH, Gliwice, Poland), Lugol's solution (I_2_/KI) was obtained from Sigma‐Aldrich, Folin–Ciocalteu reagent (Merc), sodium carbonate anhydrous (pure, POCH. Gliwice, Poland), fucoxanthin (Sigma‐Aldrich). Deionized water was filtered through qualitative filter paper (413, size: 42.5 mm, VWR) and then used to prepare solutions.

### 2.5 Total Phenolic Content

#### 2.5.1 Total Phenolic Content in Water

The total phenolic content in water was significantly lower than in the *C. glomerata* and *L. minor* extracts, with levels so minimal that they fell below the detection limit of the applied method. Consequently, the method was modified to enable the determination of phenolic content in water by using undiluted Folin–Ciocalteu reagent (Messyasz et al. 2024).

The total phenolic content in water was analyzed using a spectrophotometric method based on a modified Folin–Ciocalteu assay. For this procedure, 2 mL of ultra‐filtered habitat water, filtered through a polytetrafluoroethylene (PTFE) membrane, 0.2 μm pore size, was mixed with 150 μL of undiluted Folin–Ciocalteu reagent. After a 1‐min reaction time, 450 μL of 20% (w/v) Na_2_CO_3_ solution and 570 μL of water were added to the mixture. The samples were then incubated in the dark at 20°C for 2 h. Following incubation, 2.70 mL of the reaction mixture was transferred to a 3 mL glass cuvette, and the absorbance was measured at 760 nm using a Jenway 6400 spectrophotometer.

Standard curves created with serial dilutions of gallic acid were used for calibration. The phenol concentration was reported as milligrams of gallic acid equivalent (GAE) per liter of water. Each analysis was performed in triplicate.

The Folin–Ciocalteu method is a non‐specific assay for the determination of total phenolic content and may also react with other reducing substances, such as humic acids. To minimize this effect, all water samples were subjected to ultrafiltration, which enabled the removal of colloidal aggregates from the samples. In addition, method validation was performed. Calibration standards (gallic acid) were prepared in filtered lake water, and recovery tests were conducted by spiking this matrix with known amounts of gallic acid. Recoveries ranged from approximately 95%–100%, confirming that our modified Folin assay provides linear and reproducible results in this aqueous matrix. This procedure minimized the influence of dissolved organic matter on the measurement results; however, its contribution to the allelopathic effects cannot be completely excluded, which is a common limitation in environmental studies.

#### 2.5.2 Total Phenolic Content in *C. glomerata* Extract and *L. minor* Extract

The total phenolic content of *C. glomerata* and *L. minor* extracts was obtained using a spectrophotometric approach based on the Folin–Ciocalteu method, with slight modifications (Fabrowska et al. 2018). For the assay, 30 μL of the extract solution was combined with 1.80 mL of filtered deionized water. Subsequently, 150 μL of undiluted Folin–Ciocalteu reagent was added to the mixture. After 1 min, 450 μL of a 20% (w/v) Na_2_CO_3_ solution and 570 μL of deionized water were incorporated. The samples were incubated in the dark for 2 h at 20°C. Then, 2.70 mL of the reaction mixture was transferred into a 3 mL glass cuvette, and the absorbance was recorded at 760 nm using a Jenway 6400 spectrophotometer.

Standard curves with serial gallic acid solutions were used for calibration. The results were expressed as milligrams of gallic acid equivalent (GAE) per gram of extract. Each analysis was performed in triplicate.

### 2.6 Statistical Analysis

The ordination methods were used to establish relation between physicochemical parameters of water, phenolic content and mat size of *C. glomerata* and *L. minor* in Lake Oporzyńskie. Mat size‐physicochemical parameters of water and mat size‐phenolic content relation were explored by redundancy analysis (RDA). Due to skewed distributions of most variables, square root transformation was performed. To reduce the number of variables, a forward selection procedure using Monte Carlo test with 999 permutations was performed (variables were discarded until a significance threshold of *p* < 0.05 was reached). The relative contribution of phenolic content and other physicochemical parameters to the variation in mat size was further assessed by variance partitioning, with the significance of the resulting unique fractions tested by partial RDA with 999 permutations. Changes in mat size of *C. glomerata* and *L. minor* in response to environmental variables and months were modeled using the Generalized Additive Models (GAM) with Gamma distribution with a log link function (Hastie and Tibshirani 1990), while smooth term complexity was selected using the Akaike information criterion (Lepš and Šmilauer 2003). The physicochemical parameters were measured once per sampling, while mat size and phenolic content values were averaged across replicates to match the corresponding sampling date. All statistical analyses were performed in R version 4.6.0 (R Core Team 2026) using RStudio version 2026.05.0.218 (RStudio Team 2026), with the following packages: *vegan* (Oksanen et al. 2026) for RDA and variance partitioning, *adespatial* (Dray et al. 2026) for forward selection, *mgcv* (Wood 2011, 2017) for GAM, *DHARMa* (Hartig 2026) for residual diagnostics, and *ggplot2* (Wickham 2016) for data visualization.

## 3 Results

### 3.1 General Environmental Gradients

The water had a low phenolic content, compared to the phenolic content in the extract of *C. glomerata* (mean 12.72 mg GAE g^−1^) and *L. minor* (mean 8.16 GAE g^−1^) (Table 1). Waters containing *C. glomerata* and *L. minor* mats were characterized by high nutrient levels, increased electrolytic conductivity (mean 518 μS cm^−1^), alkaline conditions (mean pH 8.2), and high oxygen concentration (mean 8.1 mg L^−1^). Both species occurred in habitats particularly rich in ${N‐\mathrm{NO}}_{3}^{-}$ (mean 0.49 mg N L^−1^), ${N‐\mathrm{NH}}_{4}^{+}$ (mean 0.38 mg N L^−1^), and ${P‐\mathrm{PO}}_{4}^{3-}$ (mean 0.31 mg P L^−1^). Additionally, these habitats characterized by high sulphates concentration (mean 65 mg L^−1^).

**TABLE 1:** Summary statistics (minimum, maximum, mean) of phenolic content in water, in extract of *C. glomerata* and *L. minor* , mat size of *C. glomerata* and *L. minor* , and physicochemical parameters of water from May to September in 2022–2023 in Lake Oporzyńskie.

Parameter	Symbol	Unit	Minimum	Maximum	Mean
Phenolic content in water	W_phenol	mg GAE L^−1^	0.55	1.94	1.19
Phenolic content in extract of *C. glomerata*	C_phenol	mg GAE g^−1^	7.08	19.10	12.72
Phenolic content in extract of *L. minor*	L_phenol	mg GAE g^−1^	2.08	11.46	8.16
Mat size of *C. glomerata*	C_mat	m^3^	0.01	1.03	0.39
Mat size of *L. minor*	L_mat	m^3^	0.05	0.95	0.26
pH	pH	—	7.2	8.8	8.2
Temperature	Temp	°C	11.6	28.6	21.8
Total dissolved solids	TDS	mg L^−1^	265	423	361
Oxygen saturation	OS	%	25.4	103.4	70.6
Dissolved oxygen	DO	mg L^−1^	2.09	17.42	8.10
Turbidity	Turb	NTU	0.42	5.90	2.66
Electrolytic conductivity	EC	μS cm^−1^	431	642	518
Orthophosphate	${P‐\mathrm{PO}}_{4}^{3-}$	mg P L^−1^	0.05	1.04	0.31
Sulphates	${\mathrm{SO}}_{4}^{2-}$	mg L^−1^	24	73	65
Nitrate	${N‐\mathrm{NO}}_{3}^{-}$	mg N L^−1^	0.00	2.10	0.49
Ammonium nitrogen	${N‐\mathrm{NH}}_{4}^{+}$	mg N L^−1^	0.04	0.92	0.38

To examine relation between mat size of *C. glomerata* and *L. minor* and 14 environmental variables, including phenolic content and physicochemical water parameters, RDA was performed (Table 2). After forward selection (Monte Carlo test, 999 permutations), five out of 14 quantitative environmental variables were significant (*p* < 0.05), with turbidity, temperature, electrolytic conductivity, orthophosphate, dissolved oxygen, ammonium nitrogen, pH, sulphates, and nitrate showing no significant effects (*p* > 0.05).

**TABLE 2:** Results of the forward selection of environmental variables (phenolic content in water and in extract of *C. glomerata* and *L. minor* , physicochemical parameters of water) explaining variation in mat size of *C. glomerata* and *L. minor* , based on RDA. Statistical significance of selected variables was assessed with a Monte Carlo permutation test; *p* ≤ 0.05 was considered statistically significant.

Variables	*Λ*	*p*	*F*
W_phenol	0.61	**0.001**	62.16
L_phenol	0.17	**0.001**	29.77
C_phenol	0.02	**0.022**	3.99
TDS	0.03	**0.003**	6.80
OS	0.01	**0.042**	3.15
Turb	0.01	0.072	2.91
Temp	0.01	0.120	2.24
EC	0.01	0.145	2.10
${P‐\mathrm{PO}}_{4}^{3-}$	0.01	0.134	2.09
DO	0.01	0.179	1.75
${N‐\mathrm{NH}}_{4}^{+}$	0.01	0.184	1.68
pH	0.00	0.649	0.44
${\mathrm{SO}}_{4}^{2-}$	0.00	0.776	0.29
${N‐\mathrm{NO}}_{3}^{-}$	0.00	0.858	0.15

*Note:* Statistically significant values are in bold.

### 3.2 Relationship Between Phenolic Content, Physicochemical Parameters of Water and Mat Size Patterns

**FIGURE 1:**
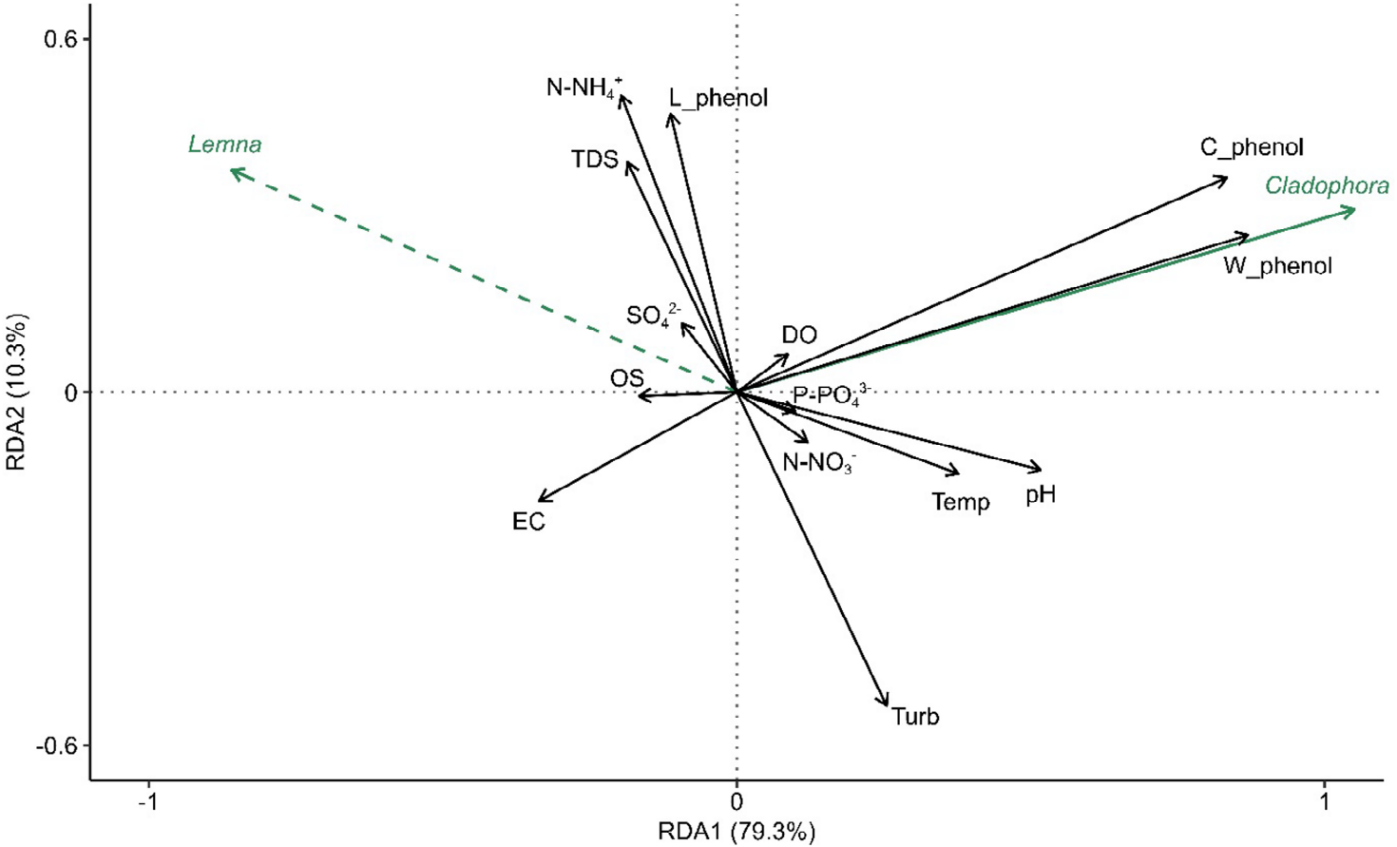
Biplot of RDA of relation between phenolic content in water, phenolic content in extract of *C. glomerata* and *L. minor* , physicochemical parameters of water and mat size of *C. glomerata* and *L. minor* . Abbreviations of the species names: *Cladophora*— *Cladophora glomerata* , *Lemna*— *Lemna minor* . Other abbreviations are described in Table 1.

The phenolic content varied depending on the sampling area in Lake Oporzyńskie and between species, with in water ranging from 0.55 to 1.94 mg GAE L^−1^ (mean 1.19 mg GAE L^−1^), while in *C. glomerata* extracts (7.08–19.10 mg GAE g^−1^, mean 12.72 mg GAE g^−1^) were higher than in *L. minor* extracts (2.08–11.46 mg GAE g^−1^, mean 8.16 mg GAE g^−1^) (Table 1). The unique fraction of phenolics accounted for an adjusted *R* ^2^ = 0.337 (*F* = 22.524, *p* = 0.001), while the unique fraction of physicochemical parameters accounted for an adjusted *R* ^2^ = 0.060 (*F* = 2.324, *p* = 0.010), both being statistically significant. A large proportion of variance (adjusted *R* ^2^ = 0.446) was jointly explained by both variable groups, and the residual fraction accounted for an adjusted *R* ^2^ = 0.157 (Tables S2 and S3). The highest explanatory power for mat size of *C. glomerata* and *L. minor* was shown by phenolic content in water (*Λ* = 0.61, *F* = 62.16, *p* = 0.001) and in extract of *L. minor* (*Λ* = 0.17, *F* = 29.77, *p* = 0.001) (Table 2). Based on RDA, mat size of *C. glomerata* was strongly positively related to high phenolic content in water and in its own extract (Figure 1). In contrast, mat size of *L. minor* was negatively related to these variables but demonstrated a weak positive relation to phenolic content in its own extract (Figure 1). The RDA revealed contrasting environmental preferences between *C. glomerata* and *L. minor* in relationship to physicochemical water parameters (Figure 1). Among the environmental variables tested in forward selection analysis, TDS (*Λ* = 0.03, *F* = 6.80, *p* = 0.003) showed the highest explanatory power for mat size of both species, followed by oxygen saturation (*Λ* = 0.01, *F* = 3.15, *p* = 0.042) (Table 2). Based on RDA, mat size of *C. glomerata* showed a moderately positive association with dissolved oxygen and orthophosphate and negative with electrolytic conductivity (Figure 1). In case of mat size of *L. minor* , there was a moderately positive association with oxygen saturation and sulphates and negative with temperature and pH (Figure 1).

**FIGURE 2:**
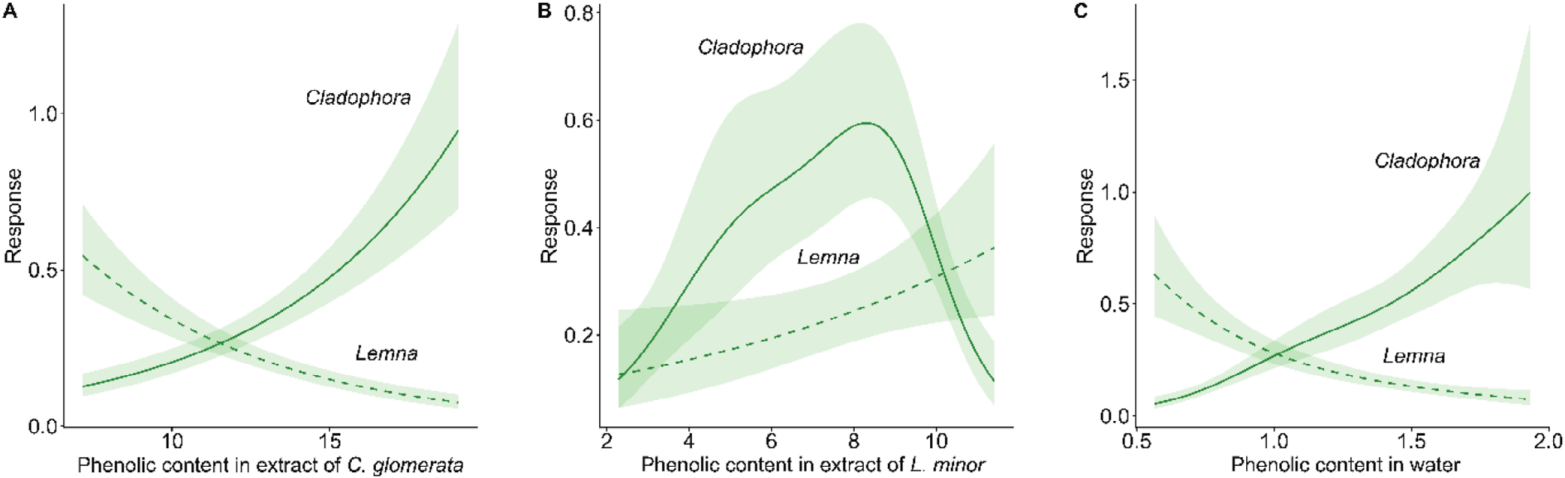
Generalized Additive Models (GAM) of the response of mat size to phenolic content A—in extract of *C. glomerata* , B—in extract of *L. minor* , and C—in water. The green bands indicate the 95% CI. Abbreviations of the species names: *Cladophora*— *Cladophora glomerata* , *Lemna*— *Lemna minor* .

The response curves (GAM model) of mat size to phenolic content in three different groups (water, *C. glomerata* extract, *L. minor* extract) were statistically significant (Table S1). Mat size of *C. glomerata* varied significantly with phenolic content in its own extract (*F* = 62.64, *p* < 0.001), showing positive associations across the content gradient, while the mat size of *L. minor* decreased with increasing phenolic content in *C. glomerata* extract (*F* = 68.50, *p* < 0.001) (Figure 2A). A similar response was observed for phenolic content in water (Figure 2C). *C. glomerata* showed a strong positive association with increasing phenolic content in water (*F* = 35.07, *p* < 0.001), while *L. minor* showed a negative response (*F* = 36.69, *p* < 0.001) (Figure 2C). A different response was observed for phenolic content in extract of *L. minor* , whose mat size showed a positive response to phenolic content in its own extract (*F* = 4.72, *p* = 0.036) (Figure 2B). Mat size of *C. glomerata* showed a strong unimodal response (*F* = 10.23, *p* < 0.001; *F* (non‐linearity) = 12.98, *p* < 0.001) (Figure 2B). Initially, these values increased, reaching peak values at intermediate levels (approximately 7–8 mg GAE g^−1^), followed by an intense decline at higher concentrations.

**FIGURE 3:**
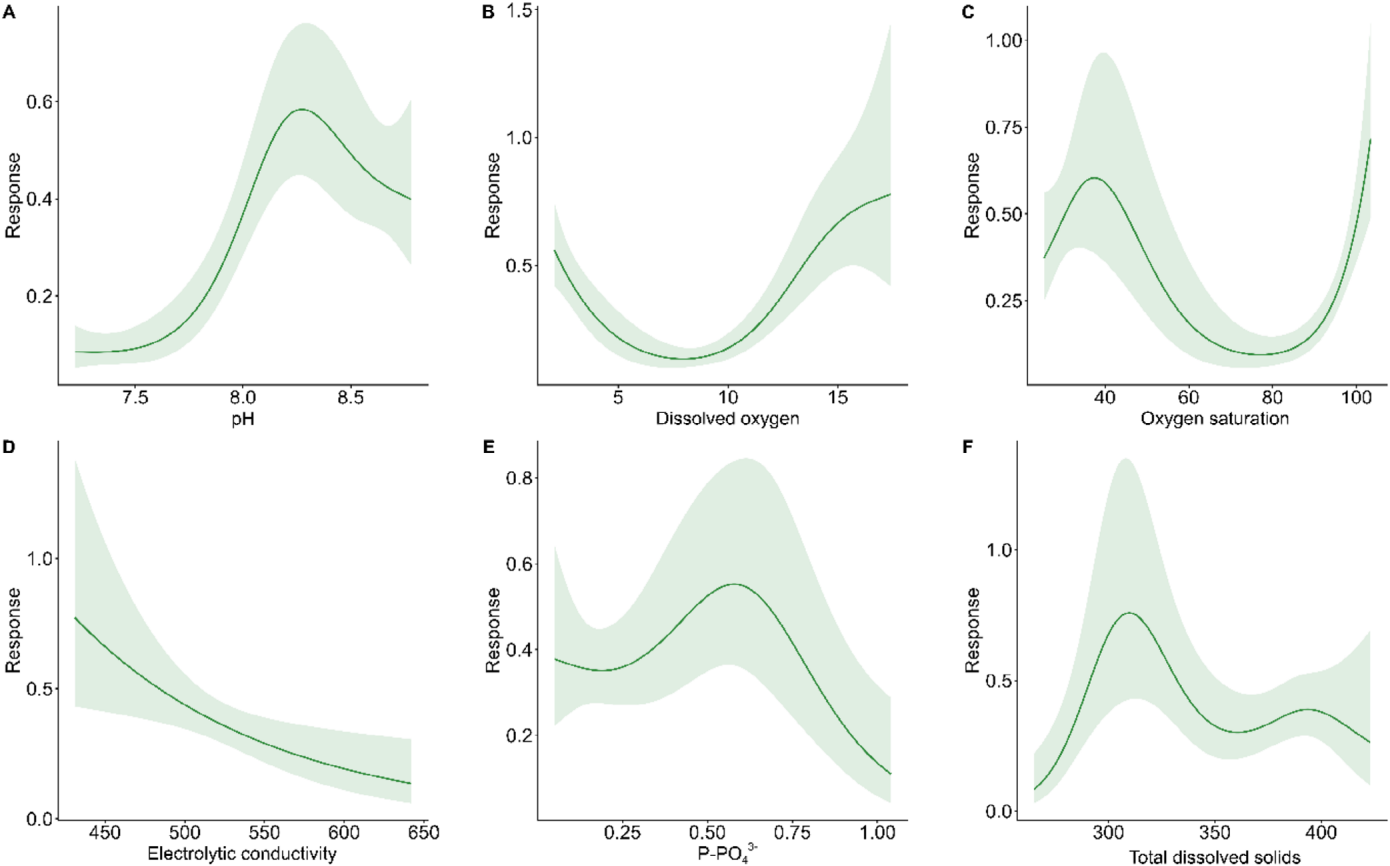
Generalized Additive Models (GAM) of the response of mat size of *C. glomerata* to: A—pH in water, B—dissolved oxygen in water, C—oxygen saturation in water, D—electrolytic conductivity, E—${P‐\mathrm{PO}}_{4}^{3-}$ in water, and F—total dissolved solids in water. The green bands indicate the 95% CI.

The response curves (GAM model) of mat size of *C. glomerata* to physicochemical parameters of water revealed significant relationships for pH, dissolved oxygen, oxygen saturation, electrolytic conductivity, orthophosphate, and total dissolved solids (Table S1, Figure 3). Mat size increased with pH up to values of approximately 8.2–8.3, above which it decreased slightly (*F* = 16.88, *p* < 0.001; *F*(non‐linearity) = 10.39, *p* < 0.001) (Figure 3A). A non‐monotonic response was observed for dissolved oxygen, with mat size being highest at low oxygen concentrations, reaching a minimum at intermediate values (8–10 mg L^−1^), and then increasing again at higher concentrations (*F* = 18.76, *p* < 0.001; *F*(non‐linearity) = 22.70, *p* < 0.001) (Figure 3B). A similar pattern was found for oxygen saturation, with mat size peaking at low saturation, decreasing to a minimum at 75%–85%, and increasing above 95% (*F* = 12.85, *p* < 0.001; *F*(non‐linearity) = 14.79, *p* < 0.001) (Figure 3C). Electrolytic conductivity showed a significant negative linear relationship with mat size (*F* = 6.62, *p* = 0.014) (Figure 3D). Mat size showed a unimodal response to orthophosphate concentration, peaking at intermediate values and decreasing at higher concentrations (*F* = 3.21, *p* = 0.032; *F*(non‐linearity) = 4.79, *p* = 0.013) (Figure 3E). Total dissolved solids showed a weakly non‐linear relationship, with mat size peaking at intermediate TDS (300–320 mg L^−1^) (*F* = 3.24, *p* = 0.033; *F*(non‐linearity) = 3.62, *p* = 0.022) (Figure 3F).

**FIGURE 4:**
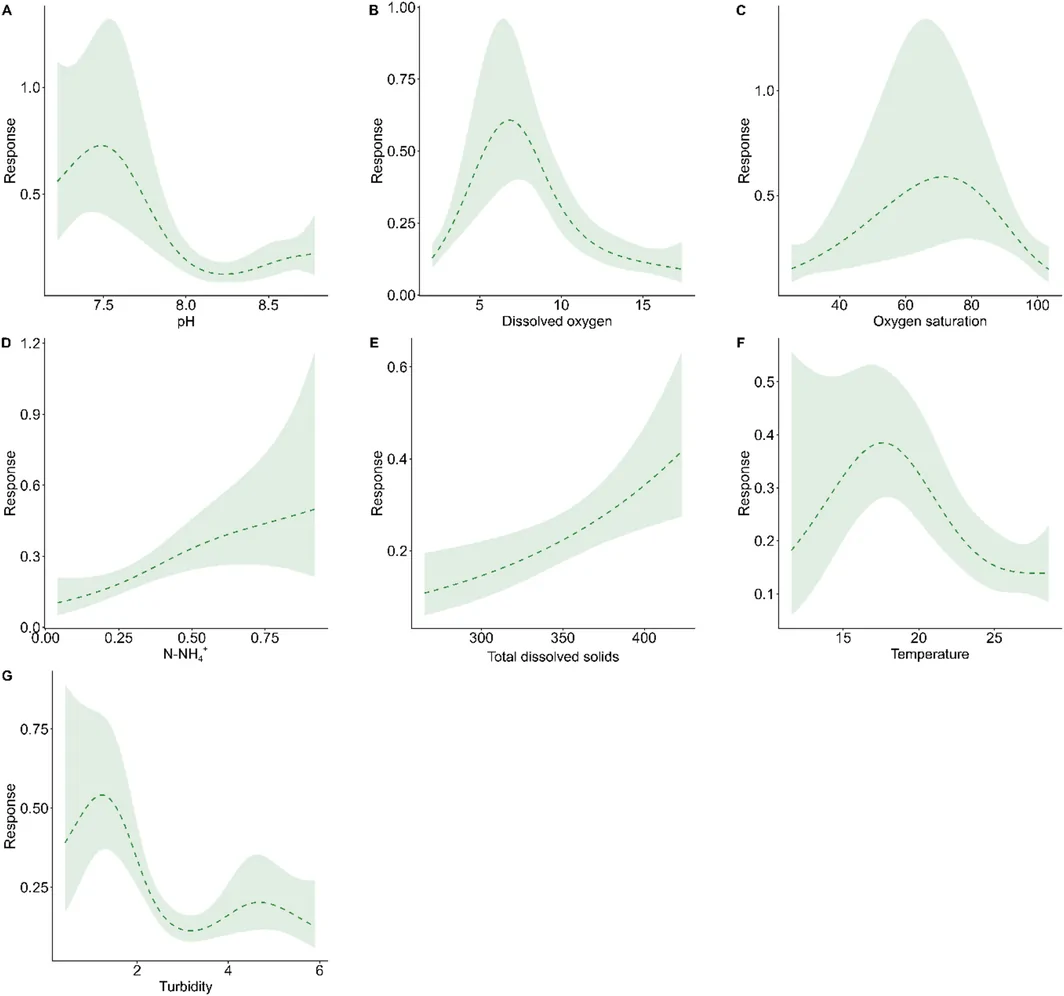
Generalized Additive Models (GAM) of the response of mat size of *L. minor* to: A—pH in water, B—dissolved oxygen in water, C—oxygen saturation in water, D—${N‐\mathrm{NH}}_{4}^{+}$ in water, E—total dissolved solids in water, F—temperature in water, G—turbidity in water. The green bands indicate the 95% CI.

For *L. minor* , significant relationships were found for pH, dissolved oxygen, oxygen saturation, ammonium nitrogen, total dissolved solids, temperature, and turbidity (Table S1, Figure 4). Mat size peaked at low pH (7.3–7.5) and declined toward higher pH values (*F* = 5.69, *p* = 0.001; *F*(non‐linearity) = 3.82, *p* = 0.018) (Figure 4A), contrasting with the pH response of *C. glomerata* . Similarly, mat size increased at low dissolved oxygen, peaked at approximately 7 mg L^−1^ and declined progressively at higher concentrations (*F* = 12.77, *p* < 0.001; *F*(non‐linearity) = 17.82, *p* < 0.001) (Figure 4B), the inverse of response observed in *C. glomerata* . For oxygen saturation, mat size showed peak response at intermediate‐to‐high values (65%–75%) (*F* = 3.16, *p* = 0.035; *F*(non‐linearity) = 5.14, *p* = 0.010) (Figure 4C). Mat size increased linearly with ammonium nitrogen (*F* = 5.14, *p* = 0.010) (Figure 4D) and similarly, it increased with total dissolved solids (*F* = 8.78, *p* = 0.005) (Figure 4E). Mat size tended to increase with temperature up to approximately 18°C and then decreased though non‐linearity was only marginal (*F* = 5.63, *p* = 0.002; *F*(non‐linearity) = 2.66, *p* = 0.073) (Figure 4F). Mat size showed a complex response to turbidity, being highest declining to a minimum around 3 NTU, and showing a small secondary peak at 4.5 NTU (*F* = 7.11, *p* < 0.001; *F*(non‐linearity) = 4.78, *p* = 0.007) (Figure 4G).

### 3.3 Seasonal Dynamics of Mat Size in Growing Season

**FIGURE 5:**
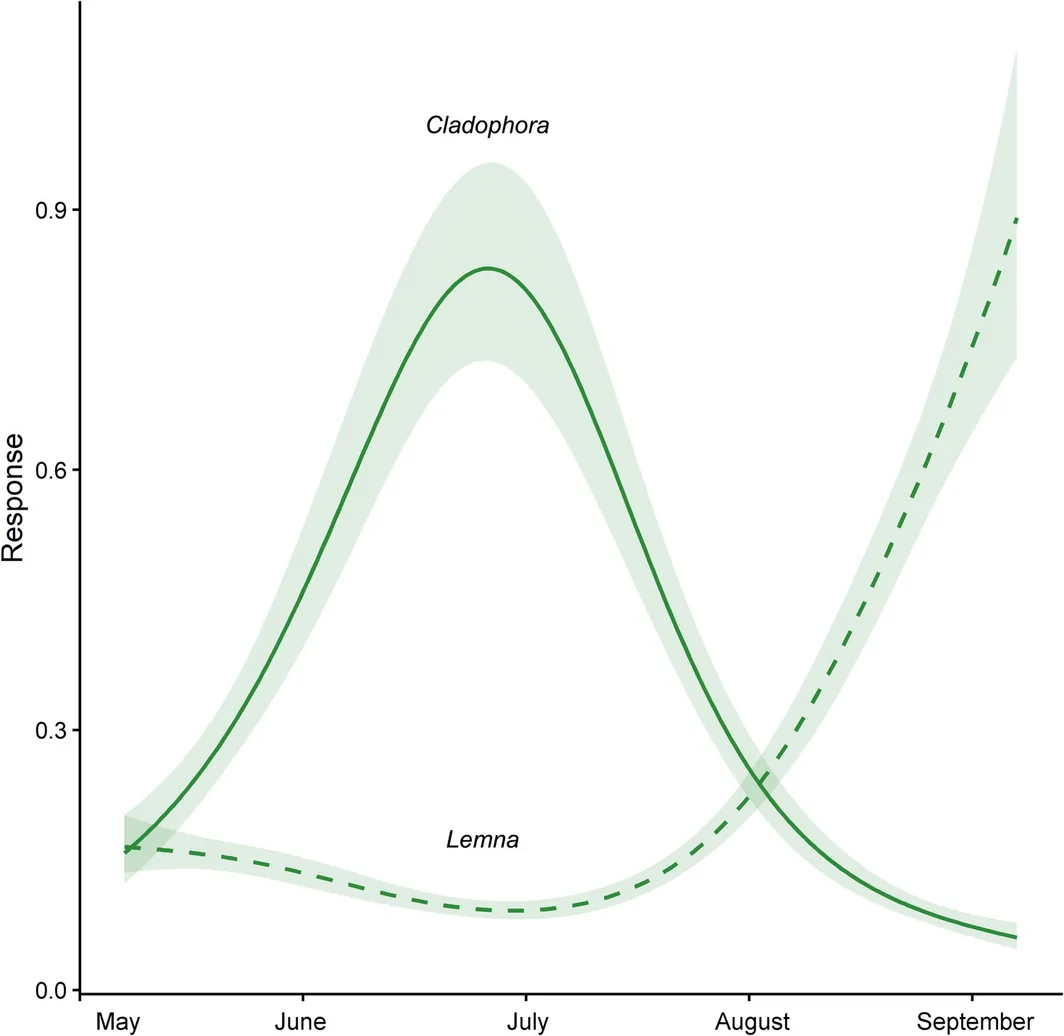
Generalized Additive Models (GAM) of the response of mat size to time in growing season. The green bands indicate the 95% CI. Abbreviations of the species names: *Cladophora*— *Cladophora glomerata* , *Lemna*— *Lemna minor* .

Mat size of *C. glomerata* ranged from 0.01 to 1.03 m^3^ (mean 0.39 m^3^), while *L. minor* mats ranged between 0.05 and 0.95 m^3^ (mean 0.26 m^3^) (Table 1). In *C. glomerata*, a characteristic unimodal seasonal pattern was observed (*F* = 123.03, *p* < 0.001; *F*(non‐linearity) = 107.16, *p* < 0.001), with mat size increasing from early spring (May), reaching maximum development during mid‐summer (July–August), and decreasing at the end of summer and the beginning of autumn (September) (Figure 5, Table S1). *L. minor* exhibited a different seasonal trajectory (*F* = 140.56, *p* < 0.001; *F*(non‐linearity) = 82.03, *p* < 0.001), with mat size showing a more gradual increase throughout the growing season and maintaining relatively high mat size from late summer to autumn (Figure 5, Table S1).

## 4 Discussion

The results of this study suggest that the seasonal dynamics of *C. glomerata* and *L. minor* mat in a eutrophic aquatic ecosystem may be simultaneously related to both allelopathic interactions and environmental gradients. Variance partitioning showed that both phenolic compounds and water physicochemical parameters together explained a larger proportion of the variance in mat size for *C. glomerata* and *L. minor* (adjusted *R* ^2^ = 0.446), exceeding either of these fractions individually. This suggests that these variables were strongly related to each other and are not independent drivers of mat size in the studied aquatic ecosystem. However, when variables were considered separately, phenolics explained uniquely a larger proportion of the variability in mat size (adjusted *R* ^2^ = 0.337) than water physicochemical parameters (adjusted *R* ^2^ = 0.060), which highlights the relatively stronger statistical association of these secondary metabolites with the patterns of *C. glomerata* and *L. minor* mat size, compared to the studied physicochemical water variables. To our knowledge, this is the first field study that has integrated analyses of chemical compounds with physicochemical water parameters in two seasons to assess their relative effect on macroalgae and macrophytes community structure. Previous research on aquatic plant communities has focused predominantly on physicochemical factors (Guo et al. 2022; Lacoul and Freedman 2006), while allelopathic interactions have been mainly studied under controlled laboratory conditions, isolating individual factors (Gross et al. 2007; van Donk and van de Bund 2002). This represents a significant knowledge gap, because allelopathic interactions are particularly important during periods of high biomass, when competition for space and resources increases (Fabrowska et al. 2018). These results are based on field observations rather than experimental studies. Therefore, we treat the relationship between phenolic compounds, the physicochemical parameters of water, and mat size as an association rather than as direct evidence that phenolic compounds inhibit or stimulate the growth of macroalgae and macrophytes.

Our results are consistent with hypothesis (i), which predicted a strong association between phenolic compounds and the size of *C. glomerata* and *L. minor* mat, with an asymmetric pattern that may reflect a competitive advantage for the macroalga. The phenolic content in water (0.55–1.94 mg GAE L^−1^) showed the strongest overall explanatory power for mat size in RDA (*Λ* = 0.61), exhibiting positive correlation with mat size of *C. glomerata* and negative correlation with *L. minor* . Additionally, the phenolic content of *C. glomerata* extracts was correlated with the phenolic content in water, suggesting that these compounds in water may originate from the macroalga. However, this source was not directly investigated in this study. This pattern is consistent with an active mechanism observed in other studies conducted in Lake Oporzyńskie, where *C. glomerata* secretes phenolic compounds into the surrounding water to achieve seasonal dominance, with secretion into the water being most pronounced during peak mat development (Fabrowska et al. 2018; Messyasz et al. 2024; Pikosz and Messyasz 2016). In contrast, *L. minor* mat size was negatively associated with phenolic content in *C. glomerata* extract, supporting the asymmetric pattern predicted by hypothesis (i). However, *L. minor* mat size responded positively to the phenolic content in its own extract, which could be consistent with the defense mechanism proposed by Fornoff and Gross 2014, in which freshwater angiosperms produce more phenolic compounds in response to biotic stress.

Although phenolic compounds were found to be stronger independent predictors, physicochemical variables contributed to a significant and independent fraction of the explained variance. This pattern was consistent with the results of GAMs, in which several physicochemical parameters, such as pH, dissolved oxygen, oxygen saturation and total dissolved solids, were identified as significant predictors for both *C. glomerata* and *L. minor* mat size. However, the direction of their reaction to these parameters was different. At the same time, some parameters such as electrolytic conductivity and orthophosphate were statistically significant only for macroalga, while parameters such as ammonium nitrogen, temperature and turbidity were statistically significant only for macrophyte. This may suggest taxon‐specific ecological preferences and varied responses to environmental factors, contributing to the different periods of dominance observed for each species during the growing season and thus confirming hypothesis (ii) of our study. For the species *C. glomerata* , the estimated optimal pH value (~8.2–8.3) is close to the value of 8.8 reported by Pikosz and Messyasz (2016), and the optimal TDS value we identified (~300–320 mg L^−1^) closely corresponds to the value of ~300 mg L^−1^ reported in the same study. Similarly, the negative relationship we found with electrolytic conductivity was directionally consistent with the unimodal optimum of ~450 μS/cm described by Pikosz and Messyasz (2016). Although in our data the relationship was linear rather than unimodal because conductivity values in our data were above this optimum, explaining why only the declining portion of the curve was observed. In contrast, the response of *C. glomerata* mat size to orthophosphate was unexpected, as mat size increased until intermediate concentrations were reached and then decreased at higher concentrations. This pattern contrasts with the positive relationship observed for similar concentration ranges by Bojorge‐García et al. (2010) and Pikosz and Messyasz (2016), which could suggest that at higher orthophosphate concentrations, other factors were unfavorable to *C. glomerata* . For *L. minor* , the pH response aligned with existing literature. Mat size peaked at ~7.3–7.5 pH, consistent with the highest growth rate reported for *L. minor* at pH 7 (Ullah et al. 2023), and contrasts with the higher pH optimum (~8.2–8.3) observed for *C. glomerata* in our study, further supporting distinct environmental preferences between the two species. In contrast, the response to temperature only partially corresponded with previous data from the literature. The size of the mat increased up to a temperature of approximately 18°C, which is consistent with reports that species of the genus Lemna tolerate a wide temperature range (11.4°C–38.1°C) (Pasos‐Panqueva et al. 2024). However, above this value the mat size was smaller, which contradicts the upper limit of this tolerance range reported in the literature. This decline may not reflect a direct negative effect of temperature on *L. minor* itself, but rather the fact that higher temperatures coincided with the peak developmental period of *C. glomerata* , whose dominance and associated competitive or allelopathic pressure may have been the actual cause of reduced *L. minor* mat size under these conditions. A similar explanation may apply to ammonium nitrogen, where the positive, linear relationship between mat size and ${N‐\mathrm{NH}}_{4}^{+}$ contrasts with reports of ammonium toxicity limiting the growth of *Lemna* species (Pasos‐Panqueva et al. 2024). The increase in ammonium concentration may simply have coincided with conditions of weakened competitive pressure from *C. glomerata* , allowing *L. minor* mats to develop more freely, independent of any direct effect of ammonium itself. This may be reinforced by the fact that ammonium toxicity is primarily attributed to the un‐ionized NH_3_ fraction, whose proportion increases with pH (Körner et al. 2003), so the low pH favored by *L. minor* in our study (~7.3–7.5) likely kept ${N‐\mathrm{NH}}_{4}^{+}$ predominantly in its less toxic ionized form.

In addition to differences in response to individual environmental factors, both species also differed in the timing of mat development during the growing season, suggesting temporal niche partitioning. As indicated by the GAM results regarding seasonal changes in mat size, *C. glomerata* dominated for most of the season, reaching a growth peak in July. Whereas, *L. minor* mats were smaller during this period, with an increase in August, when the size of the *Cladophora* mats was decreasing intensively. Given the associations between mat size, phenolic compounds, and physicochemical parameters found in these studies, it can be suggested that the growth of *L. minor* may have been limited by phenolics secreted by *C. glomerata* during its period of dominance. However, when environmental conditions became less favorable for the macroalga, its mats decreased in size, allowing the macrophyte to grow. We suggest that while phenolic compounds may create competitive asymmetry favoring the macroalga, the different physiological responses of both species to environmental gradients generate time windows in which each species can achieve dominance. Our results are consistent with previous reports that *C. glomerata* typically begins to accumulate biomass in early or mid‐spring, when its low light and low temperature preferences allow the macroalga to form mats before the main growing season (Michalak and Messyasz 2021; Pikosz et al. 2017). Biomass increases rapidly in late spring and early summer, reaching a seasonal peak between mid‐June and mid‐July, followed by a reduction in growth and population ages as water temperatures exceed approximately 22°C–23°C (Higgins et al. 2005). Similarly, literature reports on *L. minor* describe growth and biomass accumulation beginning already in spring, with biomass increasing steadily from mid‐April to mid‐June (Driever et al. 2005). Peak growth in this species is generally reported around August at the latest, as shown by photosynthetic activity and inorganic carbon uptake peaking in August (Filbin and Hough 1985), as well as by frond number and dry weight growth rates peaking at the end of that month (Arts et al. 2024). In our study, however, the marked increase in *L. minor* mat size occurred later, in August, which may be explained by competitive suppression from *C. glomerata* during its period of dominance.

This study has several limitations that should be taken into account when interpreting our results. First, although the total phenolic content in water and extracts from individual species showed strong statistical associations with mat size, this does not directly prove that phenolic compounds are the primary metabolites responsible for the observed allelopathic effect. The Folin–Ciocalteu test used in this study is a nonspecific measurement method and does not allow for the differentiation of individual phenolic compounds, nor does it exclude the participation of other secondary metabolites, which were not quantitatively analyzed in this study and may vary in combination with the content of phenolic compounds. Establishing a causal, compound‐specific link between phenolic compounds and growth inhibition would require targeted biological studies in which individual compounds would be isolated under controlled conditions. Second, our results are based on studies of a single eutrophic lake over two growing seasons, and the possibility of generalizing them to other water bodies remains to be verified. The relative importance of allelopathy compared to environmental gradients, as well as the identity of the dominant allelochemicals, may vary among lakes with different trophic status, hydrology, or aquatic plant communities. Comparative field studies involving multiple lakes, combined with controlled biological assays, are therefore necessary to determine whether phenolic compounds consistently act as the primary allelopathic agents in various aquatic ecosystems, or whether their relative importance depends on the context.

## 5 Conclusions

This study indicates that the coexistence of *C. glomerata* and *L. minor* in a eutrophic aquatic ecosystem is associated with the simultaneous effects of allelopathic interactions and environmental gradients. Allelopathic interactions mediated by phenolic compounds are potentially a stronger correlate of mat size, consistent with an asymmetric competitive relationship favoring *C. glomerata* . Physicochemical parameters of water were also significantly associated with mat size in both species, but through different variables and in different directions, reflecting distinct ecological preferences. These differing responses to environmental gradients coincide with temporal niche partitioning, with *C. glomerata* dominating during mid‐summer and *L. minor* expanding in late summer and autumn, which may facilitate coexistence despite a strong statistical association with phenolic compounds. These findings emphasize the necessity of integrating both environmental and chemical factors of competition when studying aquatic community dynamics, particularly in eutrophic systems susceptible to intense mat formation of filamentous macroalgae and free‐floating macrophytes on the water surface.

## Author Contributions

**Gostyńska Julia:** conceptualization (lead), data curation (lead), formal analysis (lead), investigation (lead), methodology (lead), project administration (lead), resources (lead), validation (lead), visualization (lead), writing – original draft (lead), writing – review and editing (lead). **Gąbka Maciej:** formal analysis (equal), methodology (equal), writing – review and editing (supporting). **Pankiewicz Radosław:** funding acquisition (equal), methodology (equal), resources (equal), writing – review and editing (supporting). **Messyasz Beata:** conceptualization (equal), funding acquisition (lead), investigation (equal), methodology (equal), project administration (equal), resources (equal), supervision (lead), validation (equal), writing – review and editing (supporting).

## Funding

This work was supported by the National Science Center, Poland—grant 2018/31/B/NZ8/00280.

## Conflicts of Interest

The authors declare no conflicts of interest.

## Supporting information


**Table S1:** Summary of generalized additive models (GAMs) for mat size of *C. glomerata* and *L. minor* in relation to environmental predictors. Models fitted with Gamma distribution and log link function. s() denotes smoothing functions; *F* and *p*‐values indicate significance of smooth terms; *F*(non‐linearity) and *p*(non‐linearity) test deviation from linearity. Abbreviations of the predictor: s(W_phenol)‐phenolic content in water, s(C_phenol)‐phenolic content in extract of *C. glomerata* , s(L_phenol)‐phenolic content in extract of *L. minor* , s(Temp)‐temperature in water, s(EC)‐electrolytic conductivity in water, s(OS)‐oxygen saturation in water, s(DO)‐dissolved oxygen in water, s(pH_‐pH in water, s(TDS)‐total dissolved solids in water, s(Turb)‐turbidity in water, s(${N‐\mathrm{NO}}_{3}^{-}$)‐nitrate in water, s(${N‐\mathrm{NH}}_{4}^{+}$)‐ammonium nitrogen in water, s(${P‐\mathrm{PO}}_{4}^{3-}$)‐orthophosphate in water, s(${\mathrm{SO}}_{4}^{2-}$)‐sulphates in water, s(Season)‐time in the growing season.
**Table S2:** Partitioning of variance in the RDA model between explanatory table X1 (phenolics) and X2 (physicochemical variables). The [a] and [b] are the unique fractions explained by X1 and X2, respectively, after accounting for the other table. The [c] is the fraction jointly explained by X1 and X2. The [d] is the unexplained (residual) fraction.
**Table S3:** Permutation tests (999 permutations) of the unique fractions of variance explained by each explanatory table, controlling for the other (partial RDA). The [a] and [b] are the unique fractions explained by X1 and X2, respectively, after accounting for the other table.

## Data Availability

The data and R code that support the findings of this study are openly available on Zenodo (Gostyńska et al. 2026, https://doi.org/10.5281/zenodo.21401947), including replicate‐level and date‐averaged measurements of mat size, phenolic content, and physicochemical water parameters, the R script used for all statistical analyses, and the associated GAM residual diagnostic plots (DHARMa). Summary statistics are additionally provided in the Appendix of this article.
