# Supplementary material for: What Drives Seasonal Mat Dynamics in Eutrophic Lake: Allelopathy or Environmental Gradients?
Source: Ecol Evol. 2026 Aug 26;16(9):e74213. doi: 10.1002/ece3.74213 (PMC13519285; doi:10.1002/ece3.74213)
Supplement: Supplementary file 1 [file ECE3-16-e74213-s001.docx]

**Appendix**

Table 1. Summary of generalized additive models (GAMs) for mat size of *C. glomerata* and *L. minor* in relation to environmental predictors. Models fitted with Gamma distribution and log link function. s() denotes smoothing functions; F and p-values indicate significance of smooth terms; F(non-linearity) and p(non-linearity) test deviation from linearity. Abbreviations of the predictor: s(W_phenol)-phenolic content in water, s(C_phenol)-phenolic content in extract of *C. glomerata*, s(L_phenol)-phenolic content in extract of *L. minor*, s(Temp)-temperature in water, s(EC)-electrolytic conductivity in water, s(OS)-oxygen saturation in water, s(DO)-dissolved oxygen in water, s(pH_-pH in water, s(TDS)-total dissolved solids in water, s(Turb)-turbidity in water, s(N-NO_3_^-^)-nitrate in water, s(N-NH_4_^+^)-ammonium nitrogen in water, s(P-PO_4_^3-^)-orthophosphate in water, s(SO_4_^2-^)-sulphates in water, s(Season)-time in the growing season.

| **Response** | **Predictor** | **Distribution** | **Link function** | **Null deviance** | **Null model residual DF** | **Deviance** | **Model residual DF** | **F** | ***p*** | **edf** | **AIC** | **F(non-linearity)** | **p(non-linearity)** |
| --- | --- | --- | --- | --- | --- | --- | --- | --- | --- | --- | --- | --- | --- |
| Mat size of *C. glomerata* | s(W_phenol) | Gamma | Log | 27.26 | 41 | 7.84 | 38.09 | 35.07 | 0.000 | 2.91 | -46.02 | 3.27 | 0.041 |
|  | s(C_phenol) | Gamma | Log | 27.26 | 41 | 11.22 | 40.00 | 62.64 | 0.000 | 1.00 | -34.33 | 0.42 | 1.000 |
|  | s(L_phenol) | Gamma | Log | 27.26 | 41 | 17.85 | 37.73 | 10.23 | 0.000 | 3.27 | -5.03 | 12.98 | 0.000 |
|  | s(Temp) | Gamma | Log | 27.26 | 41 | 25.44 | 40.00 | 3.55 | 0.067 | 1.00 | 2.59 | 0.28 | 1.000 |
|  | s(EC) | Gamma | Log | 27.26 | 41 | 24.34 | 40.00 | 6.62 | 0.014 | 1.00 | 0.40 | 0.18 | 1.000 |
|  | s(OS) | Gamma | Log | 27.26 | 41 | 15.18 | 37.52 | 12.85 | 0.000 | 3.48 | -14.99 | 14.79 | 0.000 |
|  | s(DO) | Gamma | Log | 27.26 | 41 | 12.40 | 37.83 | 18.76 | 0.000 | 3.17 | -25.25 | 22.70 | 0.000 |
|  | s(pH) | Gamma | Log | 27.26 | 41 | 12.97 | 37.58 | 16.88 | 0.000 | 3.42 | -22.62 | 10.39 | 0.000 |
|  | s(TDS) | Gamma | Log | 27.26 | 41 | 22.07 | 37.24 | 3.24 | 0.033 | 3.76 | 1.27 | 3.62 | 0.022 |
|  | s(Turb) | Gamma | Log | 27.26 | 41 | 22.64 | 38.61 | 2.40 | 0.065 | 2.39 | -0.32 | 4.64 | 0.017 |
|  | s(N-NO_3_^-^) | Gamma | Log | 27.26 | 41 | 26.61 | 39.69 | 0.40 | 0.731 | 1.31 | 5.83 | 2.12 | 0.151 |
|  | s(N-NH_4_^+^) | Gamma | Log | 27.26 | 41 | 27.22 | 40.00 | 0.09 | 0.771 | 1.00 | 5.82 | 0.35 | 1.000 |
|  | s(P-PO_4_^3-^) | Gamma | Log | 27.26 | 41 | 22.80 | 38.40 | 3.21 | 0.032 | 2.60 | 0.93 | 4.79 | 0.013 |
|  | s(SO_4_^2-^) | Gamma | Log | 27.26 | 41 | 26.81 | 39.74 | 0.28 | 0.801 | 1.26 | 5.64 | 1.78 | 0.171 |
|  | s(Season) | Gamma | Log | 27.26 | 41 | 2.86 | 37.32 | 123.03 | 0.000 | 3.68 | -87.38 | 107.16 | 0.000 |
| Mat size of *L. minor* | s(W_phenol) | Gamma | Log | 25.76 | 41 | 10.33 | 39.52 | 36.69 | 0.000 | 1.48 | -70.28 | 1.80 | 0.186 |
|  | s(C_phenol) | Gamma | Log | 25.76 | 41 | 11.09 | 40.00 | 68.50 | 0.000 | 1.00 | -68.03 | 0.15 | 1.000 |
|  | s(L_phenol) | Gamma | Log | 25.76 | 41 | 22.55 | 40.00 | 4.72 | 0.036 | 1.00 | -32.90 | 0.15 | 1.000 |
|  | s(Temp) | Gamma | Log | 25.76 | 41 | 17.03 | 38.11 | 5.63 | 0.002 | 2.89 | -45.18 | 2.66 | 0.073 |
|  | s(EC) | Gamma | Log | 25.76 | 41 | 20.74 | 38.53 | 2.90 | 0.063 | 2.47 | -34.93 | 1.99 | 0.152 |
|  | s(OS) | Gamma | Log | 25.76 | 41 | 17.31 | 38.48 | 3.16 | 0.035 | 2.52 | -37.74 | 5.14 | 0.010 |
|  | s(DO) | Gamma | Log | 25.76 | 41 | 9.75 | 37.56 | 12.77 | 0.000 | 3.44 | -66.43 | 17.82 | 0.000 |
|  | s(pH) | Gamma | Log | 25.76 | 41 | 13.93 | 37.36 | 5.69 | 0.001 | 3.64 | -48.63 | 3.82 | 0.018 |
|  | s(TDS) | Gamma | Log | 25.76 | 41 | 21.41 | 40.00 | 8.78 | 0.005 | 1.00 | -38.05 | 0.38 | 1.000 |
|  | s(Turb) | Gamma | Log | 25.76 | 41 | 14.06 | 37.16 | 7.11 | 0.000 | 3.84 | -51.40 | 4.78 | 0.007 |
|  | s(N-NO_3_^-^) | Gamma | Log | 25.76 | 41 | 24.93 | 40.00 | 1.24 | 0.272 | 1.00 | -28.72 | 0.12 | 1.000 |
|  | s(N-NH_4_^+^) | Gamma | Log | 25.76 | 41 | 20.00 | 39.33 | 5.14 | 0.010 | 1.67 | -39.92 | 1.98 | 0.166 |
|  | s(P-PO_4_^3-^) | Gamma | Log | 25.76 | 41 | 21.59 | 38.24 | 1.71 | 0.164 | 2.76 | -31.05 | 2.39 | 0.100 |
|  | s(SO_4_^2-^) | Gamma | Log | 25.76 | 41 | 24.98 | 40.00 | 1.20 | 0.280 | 1.00 | -28.39 | 0.40 | 1.000 |
|  | s(Season) | Gamma | Log | 25.76 | 41 | 1.52 | 37.14 | 140.56 | 0.000 | 3.86 | -147.31 | 82.03 | 0.000 |

Table 2. Partitioning of variance in the RDA model between explanatory table X1 (phenolics) and X2 (physicochemical variables). The [a] and [b] are the unique fractions explained by X1 and X2, respectively, after accounting for the other table. The [c] is the fraction jointly explained by X1 and X2. The [d] is the unexplained (residual) fraction.

| **Fraction** | **Df** | **R^2^** | **Adjusted R^2^** |
| --- | --- | --- | --- |
| [a+c] = X1 | 3 | 0.79907 | 0.78320 |
| [b+c] = X2 | 11 | 0.63850 | 0.50595 |
| [a+b+c] = X1 + X2 | 14 | 0.89679 | 0.84328 |
| [a] = X1 \| X2 | 3 | - | 0.33733 |
| [b] = X2 \| X1 | 11 | - | 0.06007 |
| [c] | 0 | - | 0.44588 |
| [d] = Residuals | - | - | 0.15672 |

Table 3. Permutation tests (999 permutations) of the unique fractions of variance explained by each explanatory table, controlling for the other (partial RDA). The [a] and [b] are the unique fractions explained by X1 and X2, respectively, after accounting for the other table.

| **Fraction** | **Df** | **Variance** | **F** | ***p*** |
| --- | --- | --- | --- | --- |
| [a] = X1 \| X2 (phenolics, controlling for physicochemical variables) | 3 | 0.034091 | 22.524 | 0.001 |
| Residual | 27 | 0.013622 |  |  |
| [b] = X2 \| X1 (physicochemical variables, controlling for phenolics) | 11 | 0.012898 | 2.324 | 0.010 |
| Residual | 27 | 0.013622 |  |  |
